# A qualitative exploration of work-related head injury: vulnerability at the intersection of workers’ decision making and organizational values

**DOI:** 10.1186/s12889-017-4823-5

**Published:** 2017-10-18

**Authors:** P. Kontos, A. Grigorovich, B. Nowrouzi, B. Sharma, J. Lewko, T. Mollayeva, A. Colantonio

**Affiliations:** 1 Toronto Rehabilitation Institute-University Health Network, Toronto, ON Canada; 2 Dalla Lana School of Public Health, University of Toronto, Toronto, Canada; 3 Centre for Research in Human Development, Laurentian University, Sudbury, ON Canada; 4 Rehabilitation Science Institute, University of Toronto, Toronto, Canada; 5 Laurentian University, Sudbury, ON Canada; 6 Acquired Brain Injury Research Lab, Toronto, ON Canada; 7 Department of Occupational Science & Occupational Therapy, Toronto, ON Canada

## Abstract

**Background:**

Work-related head injury is a critical public health issue due to its rising prevalence; the association with profound disruption of workers’ lives; and significant economic burdens in terms of medical costs and lost wages. Efforts to understand and prevent these types of injuries have largely been dominated by epidemiological research and safety science, which has focused on identifying risk at the level of the individual worker, population group, or organizational sector. Limited research has focused on the perspectives of the workers, a key stakeholder group for informing understanding of vulnerability to work-related head injury. This study explored workers’ perspectives to better understand their decision-making and how and why their injuries occurred.

**Methods:**

We conducted a qualitative study using in-depth semi-structured interviews with thirty-two adult workers who had sustained a work-related head injury. Workers were recruited from an urban clinic in central Ontario, Canada. Labour Process Theory informed the thematic analysis.

**Results:**

Three hazardous work conditions were identified: insufficient training; inadequate staffing; and inattention to the physical environment. In addition, professional and organizational norms were implicated in vulnerability to head injury including putting the client before the worker and the pressure to work unsafely. The findings also highlight a complex interrelationship between workers’ decision-making and professional and organizational norms that produces vulnerability to head injury, a vulnerability which oftentimes is reproduced by workers’ decisions to work despite hazardous conditions.

**Conclusions:**

Our findings suggest that, beyond the need to redress the inattention to hazards in the physical environment, there is a need to address norms that influence worker decision-making to improve the safety of workers. Using Labour Process Theory highlights an important social dynamic within workplace sectors that could inform future development and implementation of multi-level and integrated public health strategies to reduce work-related head injury.

## Background

Work-related head injury is a critical public health issue due to its rising prevalence [1]; the association with profound disruption of workers’ lives; and significant economic burdens in terms of medical costs and lost wages [2, 3]. Efforts to understand and prevent head injuries in the workplace have largely been dominated by epidemiological research that focuses on identifying risk factors predictive of traumatic brain injury [2]. Safety science is another area in which there has been progress in identifying environmental hazards (e.g. slippery floors) and worker safety behaviours (e.g. improper use of machinery) that precipitate or cause injury [4, 5]. Yet, in both epidemiological and safety science research, there is a preoccupation with ‘formal’ and ‘objective’ aspects of risk which reflects the dominance of a positivist epistemology and a view that is legitimized in functionalist forms of organizational risk analysis [6–8]. Further, the assumption in these literatures is that risk is inherent to an individual, population group [9] or a specific organizational sector, which effectively individualizes responsibility for safety by either placing the blame for injury on the individual worker [6, 7, 10, 11] or implicating poor working conditions or safety culture [12, 13]. What this polarization misses is that risk-taking may be both normative and expected as a result of organizational values and practices. Further, it omits consideration of the perspective of workers regarding how they negotiate both working tasks and safety in concert with professional and organizational norms.

Despite the call to examine workers’ decision-making regarding unsafe work practices in the context of organizational values to better understand how to support safety in the workplace [13, 14], few studies have taken this integrated approach. Labour Process Theory offers the potential to achieve a more comprehensive understanding of occupational health and safety that can be applied to workplace organization [7]. This theory describes an antagonistic dynamic of social relations between capital (e.g. managers and employers) and labour (e.g. workers) [15] that produces a continuum of possible situationally driven and overlapping worker responses ranging from resistance to accommodation, compliance, and consent [16, 17]. Labour Process Theory highlights how the “structured antagonism” [15] between workers and managers/employers results from the inequality of market relations and how this unequal social relationship both limits and structures the relations of work. The capitalist logic of accumulation drives employers/managers to control the labour process through intrinsic and extrinsic mechanisms of consent to achieve the requisite levels of performance from workers. This is the first study to explore the usefulness of this theory for exploring work-related head injury across work sectors and at the intersection of individual and organizational levels.

## Methods

The Research Ethics Board at the University Health Network approved this study, and all participants provided written informed consent.

### Design

This paper reports on qualitative findings that are part of a larger mixed-methods study of work-related head injury. The aim of the qualitative component of this larger study was to obtain insight into workers’ perspectives on how their injuries occurred, how they could have been prevented, and the subsequent accommodation they received. However, data pertaining to any accommodations are not part of our focus here.

### Participants

Participant workers who had sustained a head injury and/or had a formal diagnosis of a work-related traumatic brain injury were recruited from a neurology clinic in Ontario, Canada. The clinic is funded by the Workplace Safety and Insurance Board, a provincial organization that provides benefits and services to workers who have been injured at work or who have had an illness that is related to work. The clinic accepts assessment referrals for all work-related head injuries sustained within the province of Ontario. All participant workers were referred to the clinic for an assessment for persistent symptoms associated with a work-related head injury. No exclusion criteria were applied with respect to primary language spoken; non-English speakers were recruited and interviewed with the assistance of a certified interpreter. Based on existing research that suggests that vulnerability to injury may be gendered [18–21] and/or related to linguistic competency [22–24], we purposefully recruited to achieve equal representation of men and women and representation of diverse linguistic minorities. Our overall recruitment rate was 60%.

### Procedures

All participants were recruited by the research coordinator (BS) who provided information about the study and obtained their written informed consent. They all participated in a qualitative interview and completed a self-report questionnaire (e.g. educational history and mechanism of injury). Characteristics of the workers are summarized in Table 1. These data are presented in aggregate format by frequency of category type to ensure that participants are not identifiable.

**Table 1:** Characteristics of participants (*N* = 32)

Category	Grouping	n (%)
Sex	Female	17 (53.0)
	Male	15 (47.0)
Age Range	35–44	6 (23.0)
	45–54	9 (34.6)
	55–64	11 (42.3)
	Missing	6
Marital Status	Married/Partnered	16 (80)
	Single/Separated/Divorced/Widowed	4 (20)
	Missing	12
Education	High school or less	4 (15.4)
	Trade school or some university/college	7 (26.9)
	University or college degree	12 (46.2)
	Post-graduate	3 (11.5)
	Missing	6
Primary language spoken	English	22 (68.8)
	French	5 (15.6)
	Other	5 (15.6)
Mechanism of injury	Assault	4 (14.3)
	Fall/slip	9 (32.1)
	Motor vehicle collision	4 (14.3)
	Struck by/against object	11 (39.3)
	Missing	4
Industry/Sector	Education	5 (17.9)
	Business & Administration, Sales & Service	6 (21.4)
	Healthcare	5 (17.9)
	Manufacturing, Primary Industry	7 (25.0)
	Security, Trades & Transportation	5 (17.9)
	Missing	4

### Data collection

In-depth semi-structured qualitative interviews were conducted by a research associate (BN) who has training in qualitative interview methods and expertise in work-related head injury. The interviews were approximately 60 mins and were either conducted in person or by telephone, depending on the preference of the participant. See Table 2 for examples of questions.

**Table 2:** Examples of interview questions

Interview section	Examples of questions asked
The injury and how it occurred	Describe what happened, step by step, in as much detail as you can recall.
Training and supervision received by workers	Did you ever receive training related to your health or safety? If so, please describe what topics were covered. Were training requirements understood; and were you satisfied with the length and quality of the training you received?
Prevention of the injury	Looking back, what do you think might have prevented the injury from happening?

### Data analysis

All of the interviews were professionally transcribed, and transcripts were checked against the original recording for accuracy. We analyzed the data using a modified thematic analysis approach [25, 26] in which code development is guided by sensitizing concepts [27]; in this case, the core concepts of Labour Process Theory (e.g. structured antagonism, mechanisms of control, and consent and resistance) [16, 28]. The underlying rationalities of workers’ accounts were interpreted through an iterative process of concept building whereby we moved between our data, the central concepts of Labour Process Theory [16], and relevant research from the literature. For example, as part of our analysis of vulnerability to injury, we explored workers' decision-making and found that some workers were injured because they prioritized the safety of their client ahead of their own. Using the lens of Labour Process Theory, this was interpreted as an example of the workers’ consent to work under unsafe conditions. Further, in light of data that captured how workers’ felt compelled to place themselves in harm’s way due to their identification with organizational norms, Labour Processes Theory informed our analysis of this as managerial control that is exerted through intrinsic mechanisms*.* This was subsequently coded as ‘organizational ethos’ within the broader theme of ‘Conditions, values, and commitments.’

Four members of the research team (PK, AG, BN, BS) read the interview transcripts and two members (PK, BN) each independently coded the transcripts. The four members then discussed the codes and subsequent theme development and resolved any discrepancies in interpretation. NVivo 10 was used to facilitate electronic coding. To ensure methodological rigour and trustworthiness of the analysis, the following strategies were employed: multiple team members participated in data analysis; a dependability audit [29] was kept which included audio recordings, transcripts, interview guides, and data analysis processes and products; frequent reviews were made of the audit by the research team; and sufficient detail was provided in the audit to allow outsider assessment of theoretical generalizability [25, 30].

## Results

We first highlight hazardous work conditions which are implicated in workers’ vulnerability to head injury in the workplace. These working conditions are implicated in workers’ decisions to work in unsafe working environments. We next consider workers’ decision-making within their accounts to explore their responses to these conditions. Here we identify a continuum of possible situationally driven and overlapping worker responses that effectively produce and reproduce their continued vulnerability to head injury.

### Hazardous work conditions

The exploitative nature of how work is organized and controlled provides a critical context for understanding the vulnerability of workers to injury. In our study, workers reported limited individual autonomy to obtain the necessary skills to work safely, as well as to avoid unsafe working conditions. For example, workers described the expectation by their employers that they complete tasks they were not sufficiently trained to do. A noteworthy example is the following account by a transportation worker who suffered a head injury after his truck hit a 2'' × 2'' rock when driving underground. His training focused exclusively on surface driving and parking, and yet despite that, he was instructed to drive underground:

[I had] 4-6 hours [of] training just to show you where everything is and that’s it. After that, you’re on your own… But I was [trained on the] surface like I didn’t go underground with the truck [before]…And they were practising [with] me how to park and how to back up and all that. (Worker 901)

Workers in other sectors similarly found themselves often being instructed by their employer to do tasks without sufficient training. For example, an immigrant worker (Worker 905) from the trades sector recounted that his employer asked him to disassemble a steel floor approximately 20 ft high despite not have training to work at heights:

I don’t feel comfortable. I don’t feel comfortable with......that height…. [My supervisor]…didn’t tell me nothing [about how to work at that height]… he said... just go there... they should [have gotten] somebody [else].

Insufficient training was also found in the education sector. An education worker suffered a head injury after an autistic child in her classroom “picked up [an 8x8 inch] puzzle piece and…threw it like a Frisbee” hitting her nasal bone. When asked what might have prevented the injury she responded:

[T]here’s not enough training…there should be something…[where] you learn about autism…there should be a certain training level if you're going to have a child like that in your class…Like you can't just be thrown in there and kind of learn it on your own. (Worker 027)

Participants described how the intensification of work resulted in inadequate staffing, which precipitated their work-related head injuries. For example, a worker from the education sector, who was hit by a tetherball thrown by a student with anger management issues, cited the low worker/student ratio in her discussion of what precipitated her injury:

The support's getting less and less every year…every year it's dropping, so that we're…having to supervise these…students [with anger management issues] and then on top of that the other few hundred [students] that are out on the [school] yard…with three adults in the yard…That’s an unsafe situation. (Worker 045)

A Francophone education worker who was hit in the head by a child throwing a broom faulted government policy that sets the minimum standard for education worker/student ratios: “Normally…in Kindergarten we have two person[sic] with…30 children…but for lunch time…you…have only one…I am…opposite[sic] on this decision from the government…But have to just …obey” (Worker 902).

Insufficient staffing was not exclusive to the education sector. For example, a general labourer described how his worksite was not sufficiently staffed to ensure that he was warned about overhead work. As a result, he was struck on the head by a falling metal bar while he was stripping cement on the ground:

My company [typically]… pays people…to tell people [on the ground] ‘do not approach this area ‘cuz they are working up there’…but this company that was doing this job [above me] did not do this…so, myself and my partner, we were not aware that they were replacing those fences [above our heads]. There should always be someone in that area to …alert people [about] what’s going on. (Worker 025)

Across all of the work sectors, participants reported exposure to hazards in the physical work environment. For example, a worker from the trades sector tripped and hit his head on a ¾ inch bolt that was sticking out from the concrete at a worksite. He angrily stated: “Those bolts were all through the building… [my boss]…should have got a grinder and ground them off…because they were tripping hazards” (Worker 036). When he returned to the worksite following his hospitalization, he noted: “Somebody took a spray can, an orange spray can, and…spray painted all the bolts. And then I went back about a month later and they were all…ground off”.

Hazardous environmental conditions were also found in administrative sectors. For example, one administrative worker recounted how she was injured opening the cover of a wall cabinet above her computer: “I should have been told if you use the cabinet do not leave the lid up…if I had known [that] when I opened the cabinet I would [not have hit] my head” (Worker 041). Months after the accident, the administrative worker described how a co-worker of hers similarly experienced his cabinet door breaking and falling on his desk, and only after it narrowly missed hitting his head did their employer decide to inspect all of the office cabinets.

### Conditions, values, and commitments: The context for workers’ decision-making

Workers’ accounts of work-related head injury disclosed two prominent patterns of decision-making that were implicated in vulnerability and subsequent incidents of head injury – counter-resistance and consent. Counter-resistance was evident in many of the experiences recounted by workers where there was a strong impulse to resist unsafe working conditions that was subsequently neutralized (without explicit conflict) rather than culminating in refusal to work. For example, the immigrant worker from the trades sector who was instructed to work at heights recounted that he initially refused to do so:

[W]hen the supervisor asked me to go there I refuse[sic]…I said no, that’s not my job, because it’s not my job [But]…he said... just go there. (Worker 905)

Extrinsic factors, such as fear of reprisal were cited by some workers as the reason for their counter-resistance. The education worker who was injured by a tetherball aptly illustrates how potent this fear is. She explained that this was her second injury due to the same circumstances and that after her first injury she approached her supervisor with the request that the tetherballs be removed from the schoolyard, but her request was ignored. When asked why she continued to work despite being refused the safety measures she requested, she stated:

Most of us will always comply and look after the student first and then have to worry about our own self afterwards. And there really isn't anything in place to protect the employee. And you're really frowned upon if you refuse [the work]…They'll just ship you off to Timbuktu if you raise a stink in any way. (Worker 045)

Despite awareness of unsafe working conditions, and even awareness of the right to refuse unsafe work, the decision to continue to engage with established working arrangements and practices without any resistance was common in the workers’ accounts. In these cases, workers described how they chose to put themselves in harm’s way because of their identification with organizational values and professional commitments such as the prioritization of the needs of clients. For example, reflecting on home care management, a worker who was injured by hitting her head on a shelf in a client’s home exclaimed:

[The organization] emphasize[s] get treatment done at all costs…the emphasis is on patient care and patient safety…It’s always the [home care workers] who have a responsibility to their clients but never that…my [employer] has a responsibility to the [home care worker] to ensure their safety….It's kind of like get it done however you have to. (Worker 009)

Here, the worker is speaking of an organizational ethos of putting the client before the worker, which over time normalizes acceptance of unsafe working conditions and suppresses resistance. Indeed, when asked how she felt about working in such unsafe conditions, she said, “I guess you put up...with more than you should. It…makes you kind of numb to it… or not aware.”

This organizational ethos also appeared to be operative in the education sector where workers described sacrificing their safety in order to protect the safety of children in their care. For example, an education worker described how a student of hers had an angry outburst in class and began throwing objects, which prompted her to evacuate the other students from the class. The worker described how she remained in the classroom because of concern the student would harm himself, but then she was subsequently injured when he threw a chair that hit her head. She managed to get out of the classroom after her injury but tried to re-enter the classroom numerous times despite risking further injury:

[I thought] I can't lock him in there…so I opened the door, just to make sure he was okay…he snapped [a broom] in half and then came at me and was trying to spear me with the broom. And then I would close the door and…look in the [door] window and [could see] he was trying to spear himself in the stomach. So I would open the door and then he would try and spear me…I don't even know how long this went on. (Worker 003)

In further reflecting on the danger of the situation, she noted: "[I] thought...just...get out and, you know, save yourself. But that's not my job. I have to act as a parent would act and, you know, he was threatening to hurt himself."

## Discussion

Epidemiological and safety science research have both approached the study of workplace safety either by investigating the individual or social and organizational factors [2, 31–35]. While both fields have made progressive strides towards understanding risk factors predictive of injuries, the interrelationship between individuals and social and organizational factors remains poorly theorized and understood. Our analysis offers a new perspective on vulnerability to work-related head injury by moving beyond ‘victim blaming’ or ‘poor safety culture’ foci that result from a siloed approach. Specifically, our findings highlight a complex interrelationship between workers’ decision-making and organizational values that produces vulnerability to head injury, a vulnerability which oftentimes is reproduced by workers’ decision to continue working despite hazardous conditions. Understanding this complexity thus requires contextualizing individual experiences of head injury in relation to organizational values. Our integration of individual characteristics and organizational values in this study is precisely what Dejoy [13] suggests is necessary to ensure workplace safety management.

The majority of workers across sectors in our study expressed an awareness of the risks associated with performing their work tasks, which appears to support the assumption that workplace accidents and injuries are the consequence of unsafe worker practices [5, 36, 37]. Yet, in many cases, the accounts of workers suggest that the failure to adopt safe working practices was more a function of the organization itself failing to facilitate a safety climate. Unlike Mullen [5], who found that, despite the known risks, workers failed to adopt safe working practices implemented by their organization, we found that workers were aware of the risks but were provided neither the means nor the autonomy to conduct their work safely.

Many of the factors we identified as contributing to workers’ vulnerability to head injury can be attributed to hazardous work conditions that reflect organizational mandates that prioritize task completion at the cost of worker safety. Examples include insufficient training; inadequate supervision and staffing; and even inattention to obvious environmental safety hazards (e.g. removal of bolts in the floor). This inattention to workplace hazards suggests risks may not be appropriately regulated by health and safety standards. Ensuring the safety of workers is especially challenging in home care because the home as a workplace environment is not the standardized space of clinics and hospitals designed around professional care practices and equipment needs [38, 39]. However, it is significant that we also found environmental hazards in sectors that have received significant regulatory and empirical attention such as construction and transportation [40, 41].

Our analysis suggests that, in order to understand vulnerability to head injury in the workplace, what is needed is attention to the complex dynamic between managerial control in organizational contexts and workers’ decision-making. This dynamic highlights the importance of using Labour Process Theory to understand how decision-making is influenced by intrinsic and extrinsic mechanisms of organizational control. Extrinsic mechanisms include the progressive division and dissection of labour and progressive mechanization and automation of work, which reduces workers’ autonomy and promotes deskilling. This is exacerbated by the broader global economic restructuring of work. The introduction of lean production management systems [18, 42], with a corresponding flexible staffing model, fragmentation of skills, lone working, heavy workloads, and reduction of supervision to mere standards and technical controls, has been shown to increase the vulnerability of workers to unsafe conditions, which in turn contributes to accident and injury [18, 43]. While workers in our study were not always explicit about their reasons for counter-resistance or consent, these extrinsic mechanisms likely played a significant role in their decision-making, particularly so for immigrant workers whose more precarious labour status engenders greater fears of employer reprisal [22–24].

Intrinsic mechanisms include organizational norms that work by “managing the ‘insides’ – the hopes, fears, and aspirations – of workers, rather than their behaviors directly” [44]. In our study, attention to these mechanisms illuminated how workers’ decisions to work under unsafe working conditions was woven into the “social fabric of the organization” [45], making resistance to unsafe working conditions impossible, as this would mean challenging the organization itself [5]. In our study, workers spoke of the normative expectation in their workplace organizations that they ‘get it done however you have to’. A further example of the normative expectations we identified is the professional commitment to put the safety of clients before that of the worker – what has been termed by others as “customer sovereignty” [18] – which similarly leads to disregard for worker safety. For other workers, this pressure related to broader socio-cultural norms, specifically acting ‘as a parent would act’. These examples of intrinsic mechanisms underscore the role of organizational values and practices in the social production of vulnerability in the workplace. This echoes a concept from the medical education literature – the ‘hidden curriculum’ [46, 47] – that refers to the process of informal learning by which medical students adopt ‘cultural values and mores’ through experiences that are intrinsic to medical training institutions. This informal curriculum is often inconsistent with formal teaching and may consequently compromise workers’ learning and professional practice (e.g. prioritizing efficiency at the expense of compassionate care) [48]. In the context of our study, the ‘hidden curriculum’ was the workplace organizational norms that prioritize the safety of the client, which directly contradicts the occupational health and safety guidelines that hold workers accountable for protecting themselves from harm [49].

Understanding these complex dynamics is essential for the design and implementation of effective interventions to reduce head injury. Policy is a key level for intervention. Ontario’s workplace safety system is based on the principle of self-regulation (e.g. internal responsibility), which holds individual workers and organizations jointly accountable for ensuring safety [49]. However, in practice, it is workers themselves who are held responsible for their safety and the safety of others [50, 51]. Critiques of this system suggest that organizations should be held more accountable for ensuring a safe work environment [50, 52]. Specifically, stronger external monitoring and enforcement (e.g. greater numbers of inspectors and stricter penalties for breaching safety hazards) are deemed critical for improving organizational accountability [52, 53]. Our findings suggest that inadequate training, supervision, and staffing levels are factors that contribute to workers’ vulnerability to head injury, and, as such, determining standards for these factors and ensuring their enforcement should be important foci for external monitoring and inspection.

Further, critical education and training is needed for both employers and workers to address associations between norms and risk-taking, and, in cases where the norms themselves are implicated in work-related injury, these would need to change. For example, global McDonaldization and marketization of work [54, 55] have been linked to organizational and professional norms that prioritize the needs of clients at the expense of the safety of workers [19, 56]. Given that the prioritization of clients’ needs imbues care work with meaning [6, 56, 57], changing workers’ responses to such norms (i.e. risk-taking) is complex. Minimizing vulnerability to head injury while retaining the meaning of care work for workers, while no doubt difficult, necessitates tackling neoliberal austerity and the marketisation of social care.

### Study strengths and limitations

A common limitation cited in qualitative studies is that the findings are not generalizable because of their descriptive nature or narrow focus. However, the strength of our study is that we found a similar theoretical pattern of decision-making that produces and reproduces vulnerability to work-related head injury across a diversity of participants (e.g. across work sectors, sexes, languages spoken). This makes our findings theoretically generalizable [25].

## Conclusion

Given evidence that work-related head injury is a critical public health issue [1, 3], there is a pressing need to understand how workers participate in and reproduce conditions that they might otherwise resist. Labour Process Theory has enormous potential to help us understand the various and complex forces that shape vulnerability, which in turn can inform the development of interdisciplinary and integrated public health approaches to the development of occupational health and safety policies that better protect workers.
